# Long Non-Coding RNA LINC01123 Facilitates Cholangiocarcinoma Aggravation by Targeting miR-641

**DOI:** 10.5152/tjg.2025.24522

**Published:** 2025-01-13

**Authors:** Xueshuo Wu, Ling Wang, Xiaoming Wang, Deli Kuang, Chunmiao Yuan, Kun Xiao

**Affiliations:** 1 Department of General Surgery, The Second Affiliated Hospital of Guangzhou Medical University, Panyu Campus, Guangzhou, China; 2 Department of Infectious Diseases, Xiangya Hospital of Central South University, Changsha, China; 3 Health Management Center, Peking University Third Hospital Qinhuangdao Hospital, Qinhuangdao, China; 4 Department of Neurosurgery, Peking University Third Hospital Qinhuangdao Hospital, Qinhuangdao, China; 5 Department of Gastrointestinal Surgery, Provincial Hospital Affiliated to Shandong First Medical University, Jinan, China

**Keywords:** Cholangiocarcinoma, HCCC9810, HUCCT1, LINC01123, miR-641

## Abstract

**Background/Aims:**

Cholangiocarcinoma (CCA) is a malignant and insidious tumor that is tricky to treat. Long non-coding RNA (LncRNA) LINC01123 is a biomolecule that influences cancer progression by regulating gene expression via influencing the regulatory function of microRNAs in gene expression. Therefore, this study investigated the connection between LINC01123 and CCA and explored the underlying mechanism. The objective of this study was to provide valuable information on the management of CCA.

**Materials and Methods:**

Tumor and normal paracancer tissue samples in this study were collected from 128 CCA patients. To measure LINC01123 and miR-641 expression characteristics, quantitative reverse transcription-polymerase chain reaction was used. To explore the biological functions of LINC01123 and miR-641 in CCA cells, cell counting Kit-8 (CCK-8) and transwell migration and invasion assays were used. The mechanism was investigated using dual luciferase reporter assays and rescue experiments.

**Results:**

This study found that the expression levels of LINC01123 were higher in CCA tissues and cells than in normal tissues and cells. LINC01123 promoted the proliferation, migration and invasion ability of CCA cells and consequently became an indicator of lymph node metastasis and advanced TNM stage in CCA. Moreover, the expression of miR-641 was negatively correlated with the expression of LINC01123. LINC01123 affected CCA progression by downregulating miR-641.

**Conclusion:**

There was an upregulation of LINC01123 in CCA tumor tissues and cells. LINC01123 promoted CCA aggravation by targeting miR-641. LINC01123 could be the target of future treatment for CCA.

**Main Points**

- LINC01123 was an indicator of CCA aggravation.
- LINC01123 promotes CCA cell proliferation migration and invasion.
- miR-641 inhibits CCA cell proliferation migration and invasion.
- LINC01123 promotes CCA aggravation by regulating miR-641 expression.

## Introduction

Cholangiocarcinoma (CCA) refers to malignant tumors in the biliary system, which can be primarily categorized into intrahepatic cholangiocarcinoma (iCCA) and extrahepatic cholangiocarcinoma (eCCA).^1^ Due to CCA being insidious, most patients receive a diagnosis during the intermediate or advanced stages of the disease, which consequently reduces the survival rate of the patient.^2^ What exacerbates the situation is the lack of effective treatment methods for CCA, as immunotherapy has a limited impact on the disease.^3^ Thus, there is a desperate need for researchers to explore new and efficient methods to treat CCA and improve the overall survival rate of the disease.

Long non-coding RNAs (lncRNAs) are non-coding RNAs (ncRNAs) that contain more than 200 nucleotides in length, primarily transcribed by RNA polymerase II (Pol II).^4^ Long non-coding RNAs can control several cellular activities by altering the influence of microRNAs (miRNA) in gene expression.^5^ For instance, lncRNA indirectly inhibits the function of miRNA by binding to miRNA target genes or acting as miRNA sponges and down-regulating miRNA expression.^6^,^7^ Accordingly, lncRNAs are significantly linked to the onset and advancement of various diseases, with a particular emphasis on cancer.^6^ Long non-coding RNAs LINC01123, for example, is capable of promoting the aggravation of non-small-cell lung cancer (NSCLC) by regulating the miR-199a-5p/c-Myc axis.^8^ In CCA, LINC01123 was reported to be a potential biomarker and therapeutic target for the disease.^9^ However, the mechanism of LINC01123 in influencing CCA remains to be elucidated.

MicroRNAs are ncRNAs that contain an average length of 22 nucleotides.^10^ MicroRNAs retain fundamental biological properties in cellular activities by manipulating gene expression through binding to the 3’UTR of mRNA.^10^ Among all the miRNAs, miR-641 has been reported to be a tumor suppressor in considerable numbers of cancers, including cervical, gastric, and lung cancer.^11^^-^^13^ In CCA, miR-641 was identified as the suppressor of cancer growth and metastasis that could inhibit the expression of CDR1 Antisense RNA (CDR1as), Akt serine/threonine kinase 3 (AKT3), and mammalian target of rapamycin (mTOR).^14^ Growing evidence from numerous studies has demonstrated that miR-641 mediates the effects of various lncRNAs on cancer progression.^15^ However, there is currently no information regarding whether lncRNA LINC01123 can accelerate cancer development through the mediation of miR-641.

In this study, iCCA and eCCA patients were recruited to examine the relationship between the expression of LINC01123 and CCA. Additionally, the CCA cell lines HCCC-9810 and HUCCT1 were utilized to investigate the underlying mechanisms involved. The study posits that LINC01123 may act as a promoter of CCA and that it could enhance the progression of CCA through the regulation of miR-641. This study aimed to investigate whether LINC01123 serves as a catalyst for CCA and to elucidate the mechanisms by which LINC01123 influences this malignancy. The findings of this study provided information on the biological roles of lncRNAs in cancer, particularly in the context of CCA. Importantly, the results may also contribute to the design of new therapeutic options for CCA in the future.

## Materials and Methods

### Study Subjects

The study complied with the principles of the Declaration of Helsinki.^16^ The Ethics Committee of Peking University Third Hospital Qinhuangdao Hospital Hospital provided approval for this study (approval no. 202004706, date: July 06, 2020). A total of 128 CCA patients were enrolled in this research from 2021 to 2023 at Peking University Third Hospital Qinhuangdao Hospital Hospital. The criteria for inclusion and exclusion are delineated as follows:

1) Patients diagnosed with CCA are evaluated through the alpha-fetoprotein test, imaging studies, and biopsy procedures.
2) Patients or their immediate families were informed about the procedures of this study and signed the informed consent.
3) Patients who have not been through radiotherapy or chemotherapy were included.
4) Patients with complete follow-up, medical records, and laboratory results were included.
5) Patients with acute or chronic conditions that could bias the results were excluded.
6) Patients exhibiting severe complications, such as biliary fistula, renal failure, hepatic failure, and other malignant neoplasms, were excluded.
7) Pregnant or breastfeeding women were excluded.

### Sample Collection

The tumor and adjacent normal paracancerous tissue samples utilized in this research were obtained during surgical procedures. The collected specimens were validated by a minimum of 2 pathologists who reached a consensus regarding their classification. Subsequently, the tissues were cryopreserved using liquid nitrogen and stored at a temperature of −80°C.

### Follow-Up Survey

All patients underwent a follow-up period post-surgery ranging from 3 to 60 months to monitor their recovery and survival outcomes. The events of interest for the conclusion of the follow-up study included recurrence, metastasis, and deaths associated with CCA.

### Cell Culture

The origin of CCA cell lines included HCCC-9810 (iCCA cell line), HUCCT1 (iCCA cell line), QBC939 (eCCA cell line), and RBE (iCCA cell line), and normal intrahepatic biliary epithelial cells (HiBEC) involved in this study were obtained from the Shanghai Cell Bank of the Chinese Academy of Sciences, China. The cells were cultured in an environment of 5% CO_2_ and 37°C with RPMI 1640 medium (SH30027.02, HyClone, USA) containing 10% fetal bovine serum (FBS; SH30070.02HI, HyClone, USA) and 1% penicillin–streptomycin solution (15140122, Thermo Fisher, USA).

### Cell Transfection

In this investigation, the CCA cell lines HCCC9810 and HUCCT1 were selected for cellular-level and rescue experiments due to their heightened sensitivity to LINC01123. Before the transfection, the cells were cultured overnight in 24-well plates with RPMI 1640 medium supplemented with 10% FBS. The LINC01123 small interfering RNA (si-LINC01123; 5’-CUGAACGUCUUGCAACAGUTT-3’; siB0837165937-1-5, Ribobio, China), miR-641 mimic (miR10003311-1-5, Ribobio, China), miR-641 inhibitor (miR20003311-1-5, Ribobio, China), and corresponding negative controls (mimic-NC, inhibitor-NC, si-NC; miR1N0000001-1-5, miR2N0000001-1-5, siB06525141922-1-5, Ribobio, China; si-NC: 5’-UUCUCCGAACGUGUCACGUTT-3’) were transfected into the cells in OPTI-MEM I medium (31985070, Thermo Fisher, USA) utilizing lipofectamine 2000 (11668030, Invitrogen, USA) for a duration of 6 hours. Because the regulated target in this study was RNAs, quantitative reverse transcription-polymerase chain reaction (qRT-PCR) was used to measure the transfection efficiency in this study.

### Dual-Luciferase Reporter Assay

The binding site between miR-641 and LINC01123 was predicted using the TargetScan database. Sequences featuring either the wild-type (WT) (WT-LINC01123) or mutant (MUT-LINC01123) of LINC01123 were created and inserted into a luciferase plasmid (Gene Create company, China). The α-MEM medium (12571063, Thermo Fisher, USA) with 10% FBS was used for culturing 293T cells (Shanghai Cell Bank of the Chinese Academy of Sciences, China). After the cells achieved 80% confluence, they were seeded with the designated reporter gene constructs and the sea kidney luciferase plasmid (Gene Create company, China) into 48-well plates. A Multiskan FC (1410101, Thermo Fisher, USA) was used to measure the absorbance values of firefly and sea kidney luciferases in turn after the luciferase response substrate was added 24 hours after transfection. The ratio data were normalized using the fluorescence intensity of Renilla luciferase.

### Cell Proliferation Assay

The increase in optical density at 450 nm (OD_450_) after 0, 24, 48, and 72 hours of culture was calculated to assess cell proliferation. Cells were seeded into 96-well plates containing CCK-8 assay solution (C0038, Beyotime, China). The OD_450_ was measured after incubation at 37°C for 2 hours.

### Cell Migration Assay

For the assessment of the migratory ability of HCCC9810 and HUCCT1 cells, the Transwell migration assay (using Corning® Transwell® six well plates; CLS3450, Merck, Germany) was chosen in this study. Before the experiment, the permeable membrane was rehydrated with serum-free RPMI 1640 medium at 37°C for a duration of 30 minutes, and the cells underwent serum starvation for 12 hours. Following this, trypsin (C0201, Beyotime, China) was utilized to digest the cells, and phosphate-buffered saline (PBS; C0221G, Beyotime, China) was used to wash the digested cells twice. The cells were then resuspended in RPMI 1640 medium to achieve a concentration of 5 × 10^5^ cells/mL. The upper chamber was filled with cell suspension, while the lower chamber was filled with RPMI 1640 medium supplemented with 10% FBS, and the setup was incubated the setup for 24 hours. The Transwell chamber was washed twice with calcium-free PBS (C0221A, Beyotime, China) after the incubation. The cells were then fixed with the addition of 4% paraformaldehyde (P0099, Beyotime, China) for 30 minutes. The cells were stained with a 0.1% crystal violet solution (C0121-100 mL, Beyotime, China) for 20 minutes and washed twice with PBS to facilitate cell counting. The enumeration of cells was conducted using an optical light inverted microscope (IX71, Olympus, USA).

### Cell Invasion Assay

For the measurement of the invasive ability of HCCC9810 and HUCCT1 cells, the Transwell invasion assay was chosen in this study. In this assay, the upper chamber membrane was coated with a 50 mg/L solution of Matrigel (C0383, Beyotime, China) at a 1:8 dilution and subsequently air-dried at a temperature of 4°C.

### Total RNA Extraction

***Total RNA Extraction was Performed Utilizing TRIzol Reagent (Invitrogen, USA)***

Tissue sample RNA extraction: the tissue samples in this study were cut into 1-3 mm pieces and ground in TRIzol reagent (15596026CN, Invitrogen, USA). To dissociate the nucleic acid–protein combination, chloroform (67-66-3, Nanjing Regent, China) was added at room temperature for 10 minutes. Following a 15-minute 12 000 rpm centrifugation at 4°C, isopropanol (67-63-0, Nanjing Regent, China) was mixed with the upper aqueous phase at room temperature for 5 minutes. The RNA pellet was left at the bottom of the tube after a 15-minute 12 000 rpm centrifugation at 4°C. Then DNA contamination was cleaned out with DNase I (D7073, Invitrogen, USA). About 75% ethanol (64-17-5, Nanjing Regent, China) was utilized to wash the RNA pellet, and the mixture was centrifuged (12 000 rpm for 15 minutes) at 4°C. The RNA pellet dried in the air for 5 minutes at room temperature.

*Cell RNA extraction*: The same as tissue sample RNA extraction but without the step of grinding cells.

To determine the degree of purification and concentration, the OD_260/280_ ratio was used; high-quality RNA was defined as having a ratio between 1.8 and 2.0 and a concentration of more than 40 μg/mL.

### Real-Time Quantitative PCR

After synthesizing complementary DNA (cDNA) using the Takara PrimeScript RT Master Mix kit (RR036Q, Takara, Japan). Following this, qRT-PCR amplification of the cDNA was conducted using the Mx3000P™ Real-Time PCR System (Stratagene, Germany), incorporating specific primers for LINC01123 (forward: 5’-ACAGTGGCCGCACGCATAGCTG-3’, reverse: 5’-CTGACGACCGAGGTGACAACGATGA-3’) and miR-641 (forward: 5’-GCGCGAAAGACATAGGATAGAGT-3’, reverse: 5’-AGTGCAGGGTCCGAGGTATT-3’). The reaction parameters were established as follows: pre-denaturation: 94°C, 5 minutes; denaturation: 94°C, 20 seconds; annealing and extension: 62°C, 40 seconds; repeated for a total of 40 cycles. The mean cycle threshold (Ct) values from 3 replicate wells for each group were recorded for subsequent analysis. This study used the 2^−ΔΔCt^ method to process data.

### Statistical Analysis

The form to present the data in this study was mean ± SD. Data distributions were tested with the Shapiro–Wilk test using SPSS version 4.2.0 (SPSS Inc.; Chicago, IL, USA). To test the differences in the data from 2 groups, *t*-tests or Wilcoxon rank sum tests were used when the data followed normal distribution or abnormal distribution, respectively. To test the differences in the data from more than 2 groups, 1-way ANOVA with Tukey’s post hoc tests or Kruskal–Wallis tests with Dunnett tests were used when the data followed normal distribution or abnormal distribution, respectively. The potential of LINC01123 as a predictive biomarker for CCA progression was assessed using logistic regression analysis in SPSS version 4.2.0 (SPSS Inc.; Chicago, IL, USA). To analyze the association between clinical features and overall survival in CCA patients, the multivariate Cox proportional hazards model was calculated in SPSS (version 4.2.0). To measure the effect of LINC01123 expression on the survival probability of CCA patients in a given length of time after treatment, the Kaplan–Meier survival curve was calculated using SPSS version 4.2.0 (SPSS Inc.; Chicago, IL, USA). The association between the expression of LINC01123 and miR-641 was measured using the Pearson correlation coefficient.

## Results

### Patient Characteristics

A cohort of 128 patients diagnosed with CCA, comprising 62 men and 66 women, was included in this study, with an average age of 57 years. The CCA patient clinical characteristics are detailed in Table 1.

### Expression and Significance of LINC01123 in Cholangiocarcinoma

**Figure 1.**
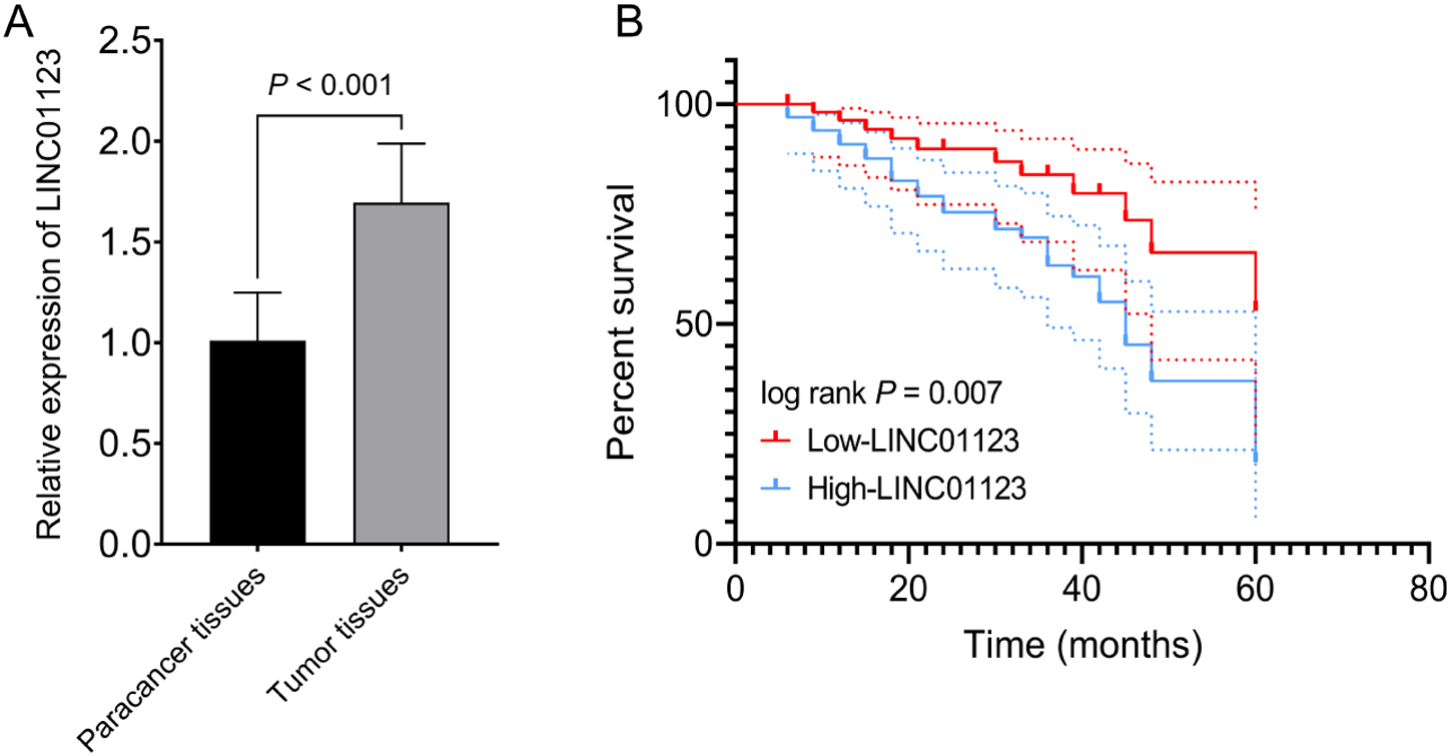
The association between LINC01123 expression and cholangiocarcinoma (CCA) and the survival probability of CCA patients. (A) The expression of LINC01123 in tumor and normal tissues in CCA patients. (B) The Kaplan–Meier survival curve based on the expression of LINC01123 in CCA patients.

The expression levels of LINC01123 were significantly higher in CCA tumor tissues compared to normal paracancer tissues (Figure 1A). The elevated level of LINC01123 (≥1.70) was an indicator of advanced TNM staging (Table 1, *P* = .025) and the presence of lymph node metastasis (Table 1, *P* = .007). Furthermore, the multivariate Cox proportional hazards model indicated a significant impact of LINC01123 expression (Table 2, HR: 2.429, 95% CI = 1.135-5.197, *P* = .022), TNM stage (Table 2, HR: 2.121, 95% CI = 1.098-4.099, *P* = .025), and lymph node metastasis (Table 2, HR: 2.610, 95% CI = 1.062-6.415, *P* = .037) on the survival time of CCA patients. Additionally, the high expression level of LINC01123 was indicative of a decreased overall survival probability among CCA patients (Figure 1B).

### Effect of LINC01123 on Cholangiocarcinoma Cells

**Figure 2.**
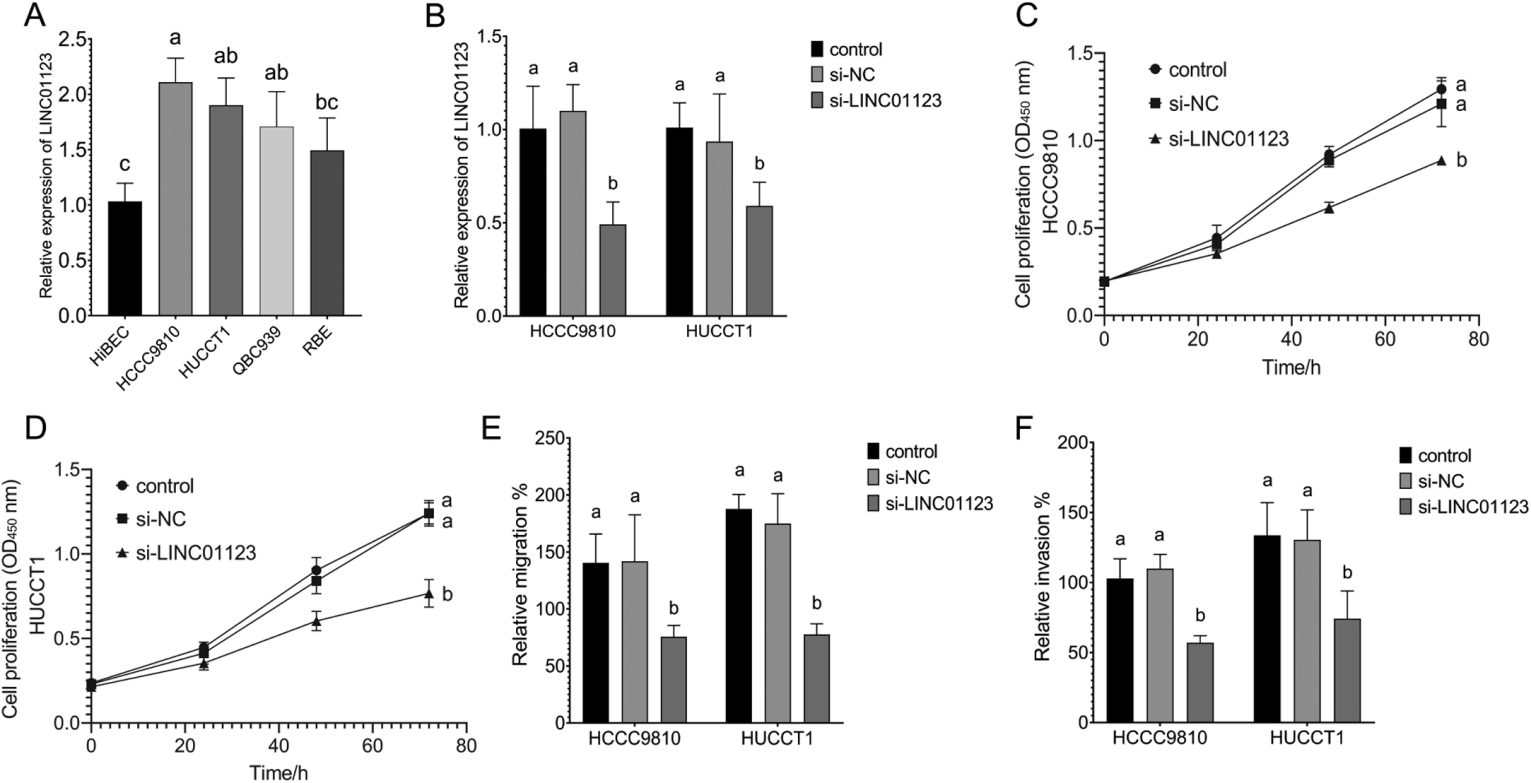
The regulatory effect of LINC01123 on CCA cell proliferation, migration and invasion. (A) The expression levels of LINC01123 in CCA cells and normal intrahepatic biliary epithelial cells. (B) The regulation of LINC01123 in CCA cells by si-LINC01123. (C-F) The effect of the expression of LINC01123 on CCA cell proliferation (C, D), migration (E), and invasion (F). Different letters represent significant differences (*P* < .05) from 1-way ANOVA with Tukey’s post-hoc test or Kruskal–Wallis test with the Dunnet test depending on the distribution characteristics of the data.

In this study, the expression of LINC01123 in both iCCA and eCCA cells was markedly higher compared to that in normal HiBEC (Figure 2A). The transfection of si-LINC01123 resulted in a significant downregulation of LINC01123 in both HCCC9810 and HUCCT1 cell lines (Figure 2B). This downregulation of LINC01123 subsequently led to a reduction in the proliferation (Figure 2C and D), migration (Figure 2E), and invasion (Figure 2F) of HCCC9810 and HUCCT1 cells.

### The Association Between LINC01123 and miR-641

**Figure 3.**
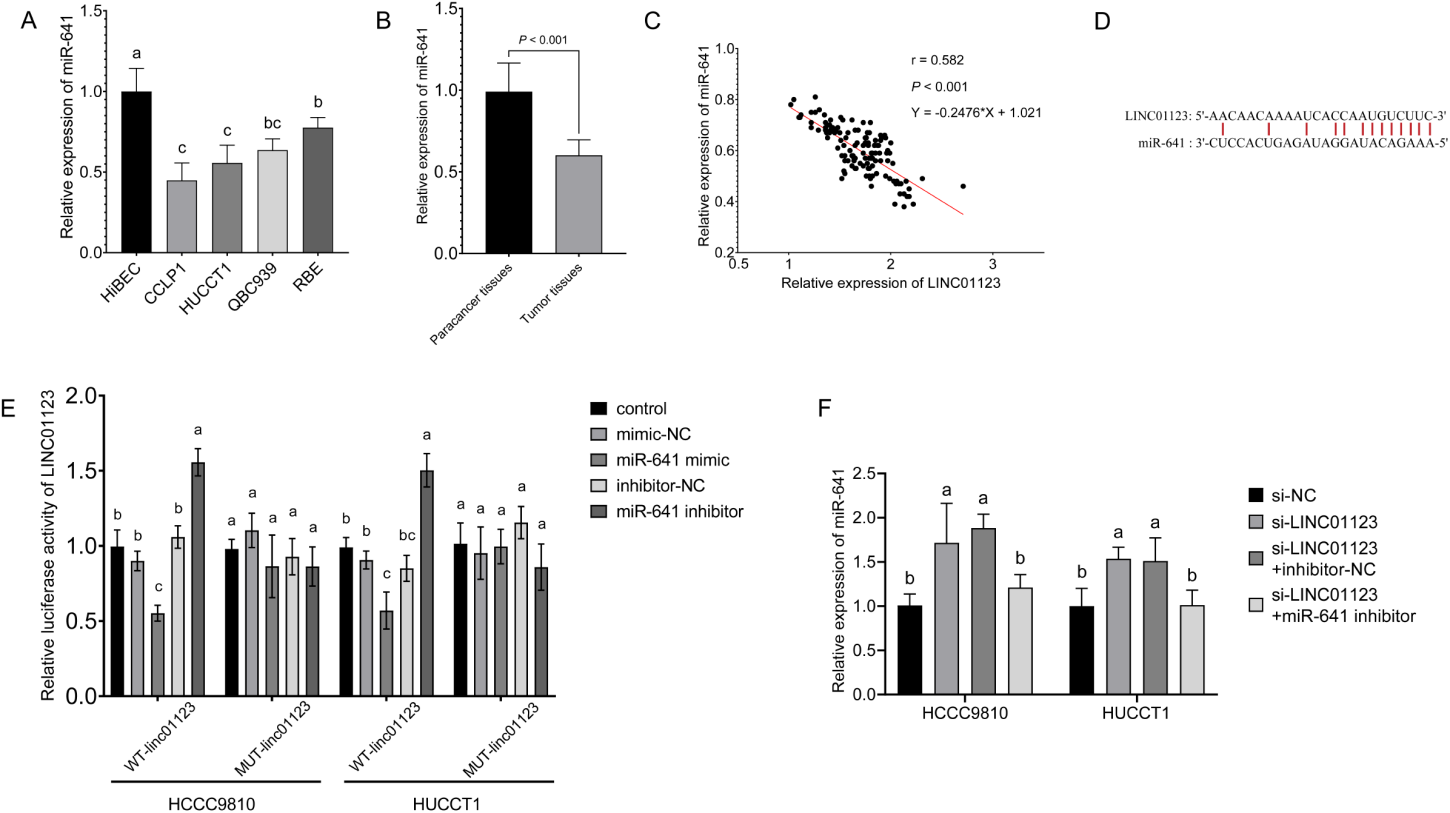
The association between LINC01123 and miR-641. (A) The expression levels of miR-641 in CCA cells and normal intrahepatic biliary epithelial cells. (B) The expression levels of miR-641 in CCA tumor tissues and normal paracancer tissues. (C and D) The regulation of miR-641 in CCA cells by miR-641 mimics and miR-641 inhibitors. (E) Pearson correlation analysis revealed the relationship between miR-641 expression and LINC01123 expression. (F) The potential binding sites between LINC01123 and miR-641. (G) Dual luciferase assay showed the luciferase activity of wild-type (WT) and mutant (MUT) LINC01123 with up- or downregulation of miR-641. (H) The expression levels of miR-641 in CCA cells regulated by si-LINC01123 and miR-641 inhibitor. Different letters represent significant differences (*P* < .05) from 1-way ANOVA with Tukey’s post-hoc test or Kruskal–Wallis test with the Dunnet test depending on the distribution characteristics of the data.

The result showed a significant reduction of miR-641 expression in CCA cells relative to normal HiBEC (Figure 3A). At the tissue level, miR-641 expression was also lower in CCA tumor tissues compared to normal paracancer tissues (Figure 3B, *P* < .001). In both HCCC9810 and HUCCT1 cell lines, the transfection of miR-641 mimics and miR-641 inhibitors resulted in a significant upregulation and downregulation of miR-641, respectively (Figure 3C and D).

An inverse relationship between miR-641 and LINC01123 expression in tumor tissues was revealed by Pearson correlation analysis (Figure 3E, *R^2^* = 0.582, *P* < .001). Utilizing TargetScan software, the potential binding site between LINC01123 and miR-641 was identified (Figure 3F). Subsequently, the luciferase reporter assay showed that the luciferase activity of WT LINC01123 (WT-LINC01123) exhibited a significant decrease upon the upregulation of miR-641 (via miR-641 mimics) and a notable increase upon the downregulation of miR-641 (via miR-641 inhibitors) in both HCCC9810 and HUCCT1 cell lines (Figure 3G). Conversely, the result did not show a significant difference between the luciferase activity of the mutant LINC01123 (MUT-LINC01123) in different groups (Figure 3G).

Rescue experiments further demonstrated that the downregulation of LINC01123 led to an upregulation of miR-641, while the transfection of miR-641 inhibitors resulted in a downregulation of miR-641 in CCA cells. These findings suggest that LINC01123 influences miR-641 expression in a manner analogous to that of miR-641 inhibitors (Figure 3H).

### LINC01123 Affects CCA Cell Behaviours by Regulating miR-641

**Figure 4.**
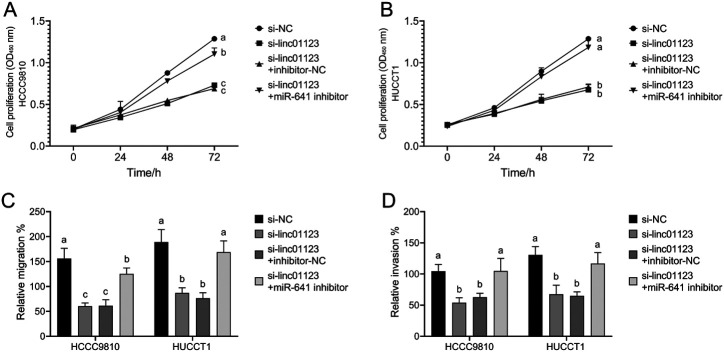
The mechanism of LINC01123 influencing CCA progression. (A-D) The rescue experiment showed that LINC01123 promoted CCA cell proliferation (A, B), migration (C), and invasion (D) by downregulating miR-641. Different letters represent significant differences (*P* < .05) from 1-way ANOVA with Tukey’s post-hoc test or Kruskal–Wallis test with the Dunnet test depending on the distribution characteristics of the data.

The rescue experiment was conducted to elucidate the mechanism by which LINC01123 influences the progression of CCA. The findings indicated that the downregulation of LINC01123 resulted in significant inhibition of CCA cell proliferation, migration, and invasion. Furthermore, the subsequent downregulation of miR-641 was found to restore the proliferative (Figure 4A and B), migratory (Figure 4C), and invasive (Figure 4D) capabilities of CCA cells. These results suggest that LINC01123 enhances CCA cell behaviors through the downregulation of miR-641.

## Discussion

Cholangiocarcinoma has received worldwide attention due to the difficulty in detecting and treating the disease and its high mortality rate.^17^ Long non-coding RNAs participate in numerous cellular activities by controlling gene expression, which makes them play an important role in cancer progression.^4^,^18^ Therefore, in this study, the connection between lncRNA LINC01123 and CCA was explored to increase the understanding of the factors that contribute to CCA progression and future therapy options. The results found an upregulation of LINC01123 in both CCA tissues and cells compared with normal biliary tissues and cells. Upregulation of LINC01123 was an indicator of advanced TNM stage, lymph node metastasis, and reduced overall survival probability of CCA patients. The results were in line with the previous study, which demonstrated that LINC01123 was upregulated in cancer tissues and promoted the malignancy of cancers.^8^ The results implied that LINC01123 was one of the key factors in encouraging CCA advancement. In recent years, the potential of lncRNAs as therapeutic targets for diseases has emerged as a clinical research hotspot.^19^ In CCA, probing methods to downregulate LINC01123 expression would be important for relieving the disease. According to previous research, potential methods that can downregulate the expression of lncRNA in cancer patients include silencing it using CRISPR/Cas13, small interfering RNA (siRNA), or antisense oligonucleotides (ASOs).^19^ This study implied that exploring CCA tumor-targeted medicine-based LINC01123-specific CRISPR/Cas13, siRNA, and ASOs might be the key to treating CCA and increasing the overall survival probability of CCA patients.

To gain detailed information on how LINC01123 promotes CCA progression, HCCC-9810 and HUCCT1 cells were involved in this study to investigate the effect of LINC01123 on CCA cellular activities. Since HCCC-9810 and HUCCT1 are essential CCA cell lines^20^ and showed a relatively high sensitivity to LINC01123, the results illustrated that the cellular activities closely related to cancer aggravation include proliferation,^21^ migration^22^,^23^ and invasion^22^,^23^ of CCA cells were inhibited by the downregulation of LINC01123. This revealed why LINC01123 upregulation was an indicator of lymph node metastasis and advanced TNM stage in CCA. Therefore, regulating LINC01123 expression levels can consequently control the rate of CCA tumor growth and metastasis. Finding a way to inhibit LINC01123 expression efficiently and specifically target CCA cells would have a chance to prolong CCA patient survival effectively. To be more specific, investigating CCA tumor-targeted medicine/therapeutic methods based on LINC01123-targeted CRISPR/Cas13 technique and LINC01123-specific siRNA and ASOs might be the hope for effectively treating CCA in the future.^19^ Moreover, considering that LINC01123 was also reported to be the regulator of cell activities in colorectal cancer^24^, hepatocellular carcinoma^25^ and colon cancer,^26^ there might be facilitating effects of LINC01123 on a wide range of cancers, not only on those that have been reported. This study further expanded the knowledge of the role LINC01123 plays in cancers and the potential of the lncRNA being the target for treating those cancers efficiently in the future.

Long non-coding RNAs participate in the cellular processes of cancers by influencing translation, mRNA stability, and serving as “sponges” for miRNAs. This study went beyond exploring the effect of LINC01123 on CCA progression and explored the miRNA that LINC01123 targeted in CCA. It was found that LINC01123 controls CCA progression by downregulating miR-641 expression. The results of this study were consistent with previous studies, which illustrated that LINC01123 can influence the progression of diseases by inhibiting the regulatory effects of specific miRNAs on gene expression.^8^ MiR-641 is an important biomolecule that participates in gene expression processes related to cancer.^27^,^28^ In CCA, miR-641 was identified as a suppressor of cancer that could inhibit the expression of cellular activity regulators including CDR1as, AKT3, and mTOR.^14^ Therefore, the information this study provided illustrated that controlling the expression levels of both LINC01123 and miR-641 would have crucial clinical significance in CCA therapy. Currently, there are 2 main forms of miRNA therapy: miRNA mimics and anti-miRNAs.^29^ MicroRNAs mimics can enhance the function of miRNAs that have been downregulated, and anti-miRNAs can silence the function of miRNAs that have been upregulated in disease.^29^ Therefore, we speculated that developing a CCA tumor-targeted miR-641 mimic is promising to suppress the disease. Moreover, considering that LINC01123 promoted CCA aggravation by downregulating miR-641 expression, interfering with the LINC01123-to-miR-641 regulatory process is also an effective treatment method for relieving CCA. Using the gene-editing technique CRISPR/Cas9 to modify the sequence of the miR-641 binding region in the LINC01123 gene may also be a potential approach for CCA treatment.^30^

Despite the crucial information on the factors influencing CCA this study provided, there were still certain limitations. Initially, the patients involved in this study came from only 1 hospital, resulting in a tiny sample size that compromised the validity of the findings. Furthermore, this study only included knockdown tests on LINC01123, with no overexpression experiments. To strengthen the credibility and rigor of the investigation, overexpression experiments should be performed simultaneously with knockdown experiments.^31^ Moreover, this study utilized only 1 siRNA, rather than 2. Although the results demonstrated that the siRNA employed in this study significantly downregulated LINC01123 expression, additional siRNAs are needed to enhance the validity of the findings. The method we used to show the success of transfection was only qRT-PCR. To ensure the success of the transfection assay, the colony formation assay and western blot should be employed in our next study. More research is needed to address the limitations of this work.

**Figure 5.**
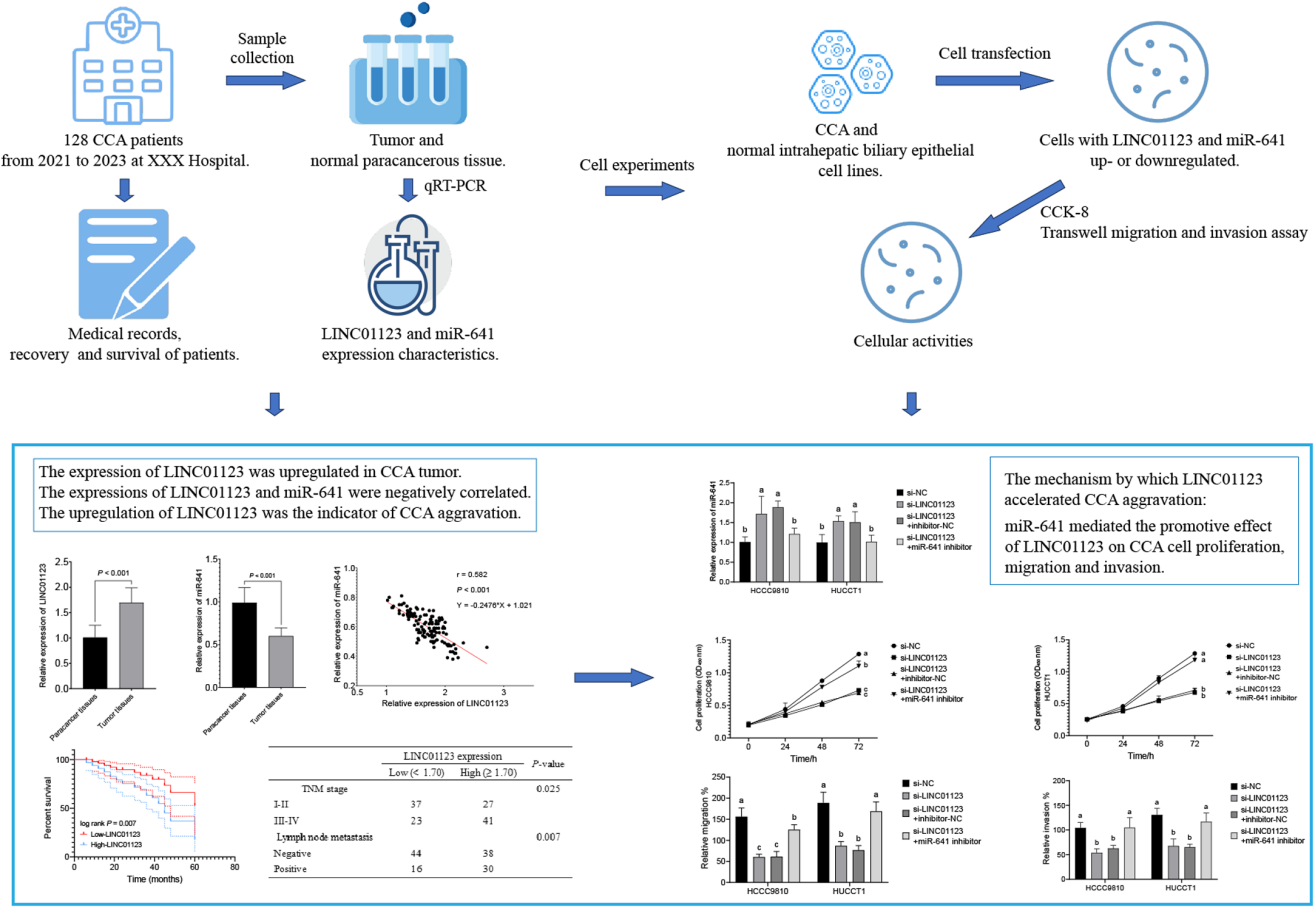
Main experimental steps and results of this study.

In conclusion, the expression of LINC01123 was higher in CCA tumors than in normal paracancer tissues. The upregulation of LINC01123 was an indicator of CCA aggravation and adverse prognosis, including lymph node metastasis and advanced TNM stage. LINC01123 could downregulate miR-641 expression. MiR-641 mediated the accelerating effect of LINC01123 on CCA aggravation. Silencing LINC01123, enhancing miR-641 functions, and interfering with the interaction between LINC01123 and miR-641 in CCA tumors could be efficient methods for treating CCA (Figure 5).

## Figures and Tables

**Table 1.** Association between LINC01123 Expression and Clinical Features of CCA Patients

	Cases (n = 128)	LINC01123 Expression		*P*
		Low (<1.70)	High (≥1.70)	
Age (years)				.141
<60	65	34	31	
≥60	63	26	37	
Sex				.406
Male	62	28	34	
Female	66	32	34	
TNM stage				.025
I-II	64	37	27	
III-IV	64	23	41	
Differentiation degree				.341
Well-moderately differentiated	90	45	45	
Poorly differentiated	38	15	23	
Location				.322
Intrahepatic	52	27	25	
Extrahepatic	76	33	43	
Lymph node metastasis				.007
Negative	82	44	38	
Positive	46	16	30	
HBV infection				.244
Negative	69	35	34	
Positive	59	25	34	

**Table 2.** Association Between Clinical Features and Overall Survival in CCA Patients

	HR Factor	95% CI	*P*
LINC01123	2.429	1.135-5.197	.022
Age	1.422	0.338-1.542	.401
Sex	1.362	0.266-1.747	.425
TNM stage	2.121	1.098-4.099	.025
Differentiation	1.174	0.022-1.366	.096
Location	1.540	0.242-1.206	.133
Lymph node metastasis	2.610	1.062-6.415	.037
HBV infection	1.585	0.280-1.244	.155

## Data Availability

The data that support the findings of this study are available on request from the corresponding author.

## References

[1] Valle JW Kelley RK Nervi B Oh DY Zhu AX. Biliary tract cancer. Lancet (London, England). 2021;397(10272):428 444. (doi: 10.1016/S0140-6736(21)00153-7) 33516341

[2] Tariq NUA McNamara MG Valle JW. Biliary tract cancers: current knowledge, clinical candidates and future challenges. Cancer Manag Res. 2019;11:2623 2642. (doi: 10.2147/CMAR.S157092) 31015767 PMC6446989

[3] Ware MB Zaidi MY Yang J Suppressive myeloid cells are expanded by biliary tract cancer-derived cytokines in vitro and associate with aggressive disease. Br J Cancer. 2020;123(9):1377 1386. (doi: 10.1038/s41416-020-1018-0) 32747748 PMC7591861

[4] Mattick JS Amaral PP Carninci P Long non-coding RNAs: definitions, functions, challenges and recommendations. Nat Rev Mol Cell Biol. 2023;24(6):430 447. (doi: 10.1038/s41580-022-00566-8) 36596869 PMC10213152

[5] Paraskevopoulou MD Hatzigeorgiou AG. Analyzing MiRNA-LncRNA interactions. Methods Mol Biol (Clifton, NJ). 2016;1402:271 286. (doi: 10.1007/978-1-4939-3378-5_21) 26721498

[6] Zhang Y Yang X Zhou L Immune-related lincRNA pairs predict prognosis and therapeutic response in hepatocellular carcinoma. Sci Rep. 2022;12(1):4259. (doi: 10.1038/s41598-022-08225-w) PMC8917134 35277569

[7] Statello L Guo CJ Chen LL Huarte M. Gene regulation by long non-coding RNAs and its biological functions. Nat Rev Mol Cell Biol. 2021;22(2):96 118. (doi: 10.1038/s41580-020-00315-9) 33353982 PMC7754182

[8] Hua Q Jin M Mi B LINC01123, a c-Myc-activated long non-coding RNA, promotes proliferation and aerobic glycolysis of non-small cell lung cancer through miR-199a-5p/c-Myc axis. J Hematol Oncol. 2019;12(1):91. (doi: 10.1186/s13045-019-0773-y) PMC6728969 31488218

[9] Li J Huang L Li Z Functions and roles of long noncoding RNA in cholangiocarcinoma. J Cell Physiol. 2019;234(10):17113 17126. (doi: 10.1002/jcp.28470) 30888066

[10] O’Brien J Hayder H Zayed Y Peng C. Overview of microRNA biogenesis, mechanisms of actions, and circulation. Front Endocrinol. 2018;9:402. (doi: 10.3389/fendo.2018.00402) PMC6085463 30123182

[11] Yao R Zheng H Wu L Cai P. miRNA-641 inhibits the proliferation, migration, and invasion and induces apoptosis of cervical cancer cells by directly targeting ZEB1. Onco Targets Ther. 2018;11:8965 8976. (doi: 10.2147/OTT.S190303) 30588009 PMC6294066

[12] Wang LW Li XB Liu Z Zhao LH Wang Y Yue L. Long non-coding RNA OIP5-AS1 promotes proliferation of gastric cancer cells by targeting miR-641. Eur Rev Med Pharmacol Sci. 2019;23(24):10776 10784. (doi: 10.26355/eurrev_201912_19780) 31858545

[13] Fan YF Yu ZP Cui XY. lncRNA colorectal neoplasia differentially expressed (CRNDE) promotes proliferation and inhibits apoptosis in non-small cell lung cancer cells by regulating the miR-641/CDK6 axis. Med Sci Monit. 2019;25:2745 2755. (doi: 10.12659/MSM.913420) 30982057 PMC6477934

[14] Li D Tang Z Gao Z Shen P Liu Z Dang X. Circular RNA CDR1as exerts oncogenic properties partially through regulating microRNA 641 in cholangiocarcinoma. Mol Cell Biol. 2020;40(15):e00042-20. (doi: 10.1128/MCB.00042-20) 32423991 PMC7364046

[15] Meng F Zhou Y Dong B Dong A Zhang J. Long non-coding RNA LINC01194 promotes the proliferation, migration and invasion of lung adenocarcinoma cells by targeting miR-641/SETD7 axis. Cancer Cell Int. 2020;20(1):588. (doi: 10.1186/s12935-020-01680-3) PMC7722326 33372601

[16] World Medical Association. World Medical Association Declaration of Helsinki: ethical principles for medical research involving human subjects. JAMA. 2013;310(20):2191 2194. (doi: 10.1001/jama.2013.281053) 24141714

[17] Banales JM Marin JJG Lamarca A Cholangiocarcinoma 2020: the next horizon in mechanisms and management. Nat Rev Gastroenterol Hepatol. 2020;17(9):557 588. (doi: 10.1038/s41575-020-0310-z) 32606456 PMC7447603

[18] Kazimierczyk M Wrzesinski J. Long non-coding RNA epigenetics. Int J Mol Sci. 2021;22(11):6166. (doi: 10.3390/ijms22116166) 34200507 PMC8201194

[19] Winkle M El-Daly SM Fabbri M Calin GA. Noncoding RNA therapeutics - challenges and potential solutions. Nat Rev Drug Discov. 2021;20(8):629 651. (doi: 10.1038/s41573-021-00219-z) 34145432 PMC8212082

[20] Isidan A Yenigun A Soma D Development and characterization of human primary cholangiocarcinoma cell lines. Am J Pathol. 2022;192(9):1200 1217. (doi: 10.1016/j.ajpath.2022.05.007) 35640676 PMC9472155

[21] Gaglia G Kabraji S Rammos D Temporal and spatial topography of cell proliferation in cancer. Nat Cell Biol. 2022;24(3):316 326. (doi: 10.1038/s41556-022-00860-9) 35292783 PMC8959396

[22] Novikov NM Zolotaryova SY Gautreau AM Denisov EV. Mutational drivers of cancer cell migration and invasion. Br J Cancer. 2021;124(1):102 114. (doi: 10.1038/s41416-020-01149-0) 33204027 PMC7784720

[23] Krakhmal NV Zavyalova MV Denisov EV Vtorushin SV Perelmuter VM. Cancer invasion: patterns and mechanisms. Acta Nat. 2015;7(2):17 28. (doi: 10.32607/20758251-2015-7-2-17-28) PMC4463409 26085941

[24] Liu Z Ma L Gu Y Long non-coding RNA LINC01123 promotes cell proliferation, migration and invasion via interacting with SRSF7 in colorectal cancer. Pathol Res Pract. 2022;232:153843. (doi: 10.1016/j.prp.2022.153843) 35325644

[25] Xiao Z Liu Y Zhao J Long noncoding RNA LINC01123 promotes the proliferation and invasion of hepatocellular carcinoma cells by modulating the miR-34a-5p/TUFT1 axis. Int J Biol Sci. 2020;16(13):2296 2305. (doi: 10.7150/ijbs.45457) 32760198 PMC7378647

[26] Ye S Sun B Wu W LINC01123 facilitates proliferation, invasion and chemoresistance of colon cancer cells. Biosci Rep. 2020;40(8):BSR20194062. (doi: 10.1042/BSR20194062) 32700743 PMC7414518

[27] Li L Wei D Zhang J Deng R Tang J Su D. miR-641 inhibited cell proliferation and induced apoptosis by targeting NUCKS1/PI3K/AKT signaling pathway in breast cancer. Comp Math Methods Med. 2022;2022:5203839. (doi: 10.1155/2022/5203839) PMC8769837 35069784

[28] Avsar R Gurer T Aytekin A. Bioinformatics and expression analyses of miR-639, miR-641, miR-1915-3p and miR-3613-3p in colorectal cancer pathogenesis. J Cancer. 2023;14(13):2399 2409. (doi: 10.7150/jca.86974) 37670968 PMC10475367

[29] Kim T Croce CM. MicroRNA: trends in clinical trials of cancer diagnosis and therapy strategies. Exp Mol Med. 2023;55(7):1314 1321. (doi: 10.1038/s12276-023-01050-9) 37430087 PMC10394030

[30] S Zibitt M Hartford CCR Lal A. Interrogating lncRNA functions via CRISPR/Cas systems. RNA Biol. 2021;18(12):2097 2106. (doi: 10.1080/15476286.2021.1899500) 33685382 PMC8632070

[31] Moriya H. Quantitative nature of overexpression experiments. Mol Biol Cell. 2015;26(22):3932 3939. (doi: 10.1091/mbc.E15-07-0512) 26543202 PMC4710226

